# Supplementary material for: Single-nucleotide polymorphisms of uracil-processing genes affect the occurrence and the onset of recurrent depressive disorder
Source: PeerJ. 2018 Jun 26;6:e5116. doi: 10.7717/peerj.5116 (PMC6025148; doi:10.7717/peerj.5116)
Supplement: Table S1 — Table presents a distribution of genotypes and alleles of DUT rs4775748, SMUG rs3087404 and UNG rs34259 single-nucleotide polymorphisms, and OR with 95% CI in group of patients with rDD that had their first episode at or after 35 years of age (marked as late onset depression) and group of patients with rDD that had their first episode at or after 35 years of age (marked as late onset depression). [file peerj-06-5116-s002.docx]

| Genotype  /Allele | Late onset depression  (n = 125) | | Early onset depression  (n = 127) | | Crude OR (95% CI) | *p* | Adjusted OR (95% CI) | *p* |
| --- | --- | --- | --- | --- | --- | --- | --- | --- |
|  | Number | Frequency | Number | Frequency |  |  |  |  |
| *DUT* (rs4775748) | | | | | | | | |
| T/T | 94 | 0.752 | 92 | 0.724 | 0.867 (0.494-1.521) | 0.619 | 0.829 (0.470-1.463) | 0.518 |
| T/G | 27 | 0.216 | 31 | 0.244 | 1.172 (0.651-2.110) | 0.596 | 1.242 (0.685-2.253) | 0.475 |
| G/G | 4 | 0.032 | 4 | 0.031 | 0.984 (0.241-4.023) | 0.982 | 0.937 (0.227-3.862) | 0.929 |
| *χ^2^* = 0.282; *p* = 0.869 | | | | | | | | |
| T | 215 | 0.860 | 215 | 0.846 | 0.905 (0.562-1.455) | 0.680 | 0.882 (0.546-1.424) | 0.607 |
| G | 35 | 0.140 | 39 | 0.154 | 1.105 (0.687-1.778) | 0.680 | 1.134 (0.702-1.832) | 0.607 |
| *SMUG1* c.-31A>G(rs3087404) | | | | | | | | |
| A/A | 36 | 0.288 | 26 | 0.205 | 0.636 (0.357-1.136) | 0.126 | 0.587 (0.325-1.060) | 0.077 |
| A/G | 63 | 0.504 | 66 | 0.520 | 1.065 (0.650-1.745) | 0.803 | 1.099 (0.668-1.809) | 0.710 |
| G/G | 26 | 0.208 | 35 | 0.276 | 1.449 (0.810-2.591) | 0.212 | 1.489 (0.829-2.675) | 0.183 |
| *χ^2^* = 2.995; *p* = 0.224 | | | | | | | | |
| A | 135 | 0.540 | 118 | 0.465 | 0.732 (0.512-1.047) | 0.088 | 0.702 (0.488-1.010) | 0.057 |
| G | 115 | 0.460 | 136 | 0.535 | 1.366 (0.955-1.953) | 0.088 | 1.425 (0.990-2.049) | 0.057 |
| *UNG* (rs34259) | | | | | | | | |
| G/G | 63 | 0.504 | 51 | 0.402 | 0.660 (0.401-1.088) | 0.103 | 0.675 (0.409-1.115) | 0.125 |
| G/C | 53 | 0.424 | 66 | 0.520 | 1.470 (0.894-2.416) | 0.129 | 1.418 (0.859-2.340) | 0.172 |
| C/C | 9 | 0.072 | 10 | 0.079 | 1.102 (0.432-2.810) | 0.839 | 1.168 (0.454-3.000) | 0.748 |
| *χ^2^* = 2.720; *p* = 0.257 | | | | | | | | |
| G | 179 | 0.716 | 168 | 0.661 | 0.752 (0.503-1.124) | 0.164 | 0.756 (0.506-1.131) | 0.173 |
| C | 71 | 0.284 | 86 | 0.339 | 1.330 (0.890-1.986) | 0.164 | 1.322 (0.884-1.978) | 0.173 |

* OR adjusted for sex.

*p* < 0.05 along with corresponding ORs are in bold
